# Dual-Responsive Nanotubes Assembled by Amphiphilic Dendrimers: Controlled Release and Crosslinking

**DOI:** 10.3390/ma13163479

**Published:** 2020-08-07

**Authors:** Minghui Zhang, Hui Yang, Jiazhong Wu, Siyu Yang, Danfeng Yu, Xu Wu, Aiqing Ma, Keji Sun, Jinben Wang

**Affiliations:** 1 CAS Key Lab of Colloid, Interface and Chemical Thermodynamics, Institute of Chemistry, Chinese Academy of Sciences, Beijing 100190, China; zhangminghui@iccas.ac.cn (M.Z.); jbwang@iccas.ac.cn (J.W.); 2 University of Chinese Academy of Sciences, Beijing 100049, China; 3 State Key Laboratory of Enhanced Oil Recovery, Research Institute of Petroleum Exploration and Development of PetroChina, Beijing 100083, China; wujiazhong@petrochina.com.cn (J.W.); yangsiy@petrochina.com.cn (S.Y.); 4 Department of Chemistry and Chemical Engineering, Guangzhou University, Guangzhou 510006, China; ccyudanfeng@gzhu.edu.cn (D.Y.); xuwu@gzhu.edu.cn (X.W.); 5 Petroleum Engineering Technology Research Institute, Shengli Oilfield Branch, China Petrochemical Corporation LTD, Dongying 257000, China; maq7979@163.com (A.M.); skj502@sina.com (K.S.)

**Keywords:** amphiphilic dendrimer, LbL assembly, dual-responsive nanocapsule, controllable release and gelation

## Abstract

Although stimuli-responsive release systems have attracted great attention in medical applications, there has been no attempt at “precise” deep profile control based on such systems, which is greatly need to improve oil recovery. With this in mind, we provided a facile and simple strategy to prepare stimuli-responsive composite capsules of amphiphilic dendrimers–poly(styrene sulfonic acid) sodium/halloysite nanotubes (HNTs) via layer-by-layer (LbL) self-assembly technique, controlling the release crosslinking agent methenamine under different pH or salinity conditions. The release time of methenamine encapsulated in multilayer shells is about 40 h, which can be prolonged with the introduction of salt or shortened via the addition of acid, which accordingly induces the gelation of polyacrylamide (PAM) solutions, taking from a few hours to a dozen days. This study provided a novel approach for controllable release of chemical agents and controllable crosslinking of deep profiles in many application fields.

## 1. Introduction

Stimuli-responsive delivery devices, known as “smart” release nanosystems, are poised to be used in diverse applications such as tumor therapy, antisepsis, and corrosion protection, which require tuning release via external stimuli [1,2,3]. Most recently, controllable release systems have been desired in practical application fields such as the petroleum industry due to the great need for “precise” profile control in order to enhance oil recovery [4,5,6,7]. After long-term water flooding, the vertical heterogeneity of a reservoir’s porosity is dramatically enlarged, resulting in increased water outflow and decreased crude oil production. Deep control seems to be a promising way to solve the problem by improving the efficiency of the water sweeping process into the middle-low permeability reservoir, thus enhancing oil recovery. The traditional method in profile control is that the mixing fluid, composed of polyacrylamide and a crosslinking agent, is injected without “precise” control of the gelation time, causing a blockage near the bottom of the well and reducing the water flooding efficiency. The ideal gelation time should be able to be regulated over a wide range and selected according to the real oil field conditions, for which problems still remain due to unpredictable gelation positions [8]. Stimuli-responsive nanocapsules are proposed as a potential candidate system to realize precise deep control and improve the efficiency of oil production through controlling the releasing rate of the crosslinking agent and correspondingly regulating the gelation time and position [9]. The prepolymer is crosslinked with the crosslinking agent released by the nanocapsules when they reach the expected position, and the crosslinked polymer with increased viscosity moves forward with the injected water, blocking the flow channel of the high-permeability reservoir and changing the flow direction of the injected reservoir. The sweep coefficient of the injected water can be greatly improved, thus improving the oil recovery. Currently, such research and technology are still lacking, due to missing materials and insufficient understanding of the related stimuli-responsive mechanisms.

Amphiphilic dendrimers, as an important branch of the dendrimer family, can be endowed with amphiphilic balance and unique aggregation behavior by introducing appropriate modifications [10,11,12,13,14,15]. Compared with conventional dendrimers, amphiphilic ones show a talent for forming various self-assembled structures on solid surfaces, which may respond to external stimuli, such as the pH and ionic strength [16,17]. In our previous study, we designed a series of alkyl-terminated cationic poly(amidoamine) dendrimers with different generations, exhibiting flexible supramolecular self-assembly at different salinity levels [18,19]. It can be predicted that a smart nanocontainer based on such dendrimer systems may possess perfect environmentally sensitive functions. With this in mind, we have fabricated a series of responsive layer-by-layer (LbL) assemblies, which were assembled via electrostatic interaction between cationic amphiphilic dendrimers and anionic poly(styrene sulfonic acid) sodium (PSS) and which were used to modify halloysite nanotubes (HNTs) as cargo delivery in this study. The methenamine release behavior was shown to be ideal in such nanosystems, covering a wide range from a couple of hours to a dozen days at different salinity or pH values, while the polyacryamide (PAM) crosslinking performance was further carried out under control. In practical applications, the gelation time and strength can be manipulated precisely through adjusting the pH and salinity of the injected water according to the specific reservoir conditions. To the best of our knowledge, this is the first time this kind of dual-responsive nanocapsule system has been developed and the controllable release mechanism has been explored, which is expected to provide guidance and a reference for environmentally responsive, low-cost, environmentally friendly delivery systems in application fields.

## 2. Experimental Section

### 2.1. Materials

Halloysite nanotubes (HNTs) were purchased from the Guangzhou Runwo Materials Technology Co., Ltd. (Guangzhou, China), followed by fine grinding and purification according to previous reports [20,21,22,23]. Amphiphilic dendrimers G_n_C_12_ (n = 1, 2, 3) were synthesized as shown in Scheme S1 and the detailed characterization results are exhibited in Figures S1–S3. NaCl solution was selected to explore the salinity responsiveness of LbL assemblies, because NaCl is a commonly used inorganic salt in injected water in oil fields. Poly (styrene sulfonic acid) sodium (PSS, *M*_w_ ≈ 70000), NaCl, HCl, and methenamine were all purchased from Alfa Aesar (Ward Hill, MA, USA). Deionized water was used for all the experiments.

### 2.2. Methods

The adsorption and responsiveness of LbL assemblies onto solid surfaces were achieved using a Q-Sense E1 instrument (Biolin Scientific AB, Gothenburg, Sweden). Silica coated AT-cut quartz crystals (QSX 301, Biolin Scientific AB, Gothenburgcity, Sweden) with a resonant frequency of 4.95 MHz were selected to represent HNT surfaces, due to the similar amount of hydroxyl groups at the surface. The quartz crystals were cleaned by plasma treatment using an O_2_-plasma etcher for 15 min, followed by an immersion in 2 vol% sodium dodecyl sulfate in water for 10 min, rinsing with Milli-Q water, drying with nitrogen, and a final O_2_-plasma treatment for 10 min. The temperature for all the quartz crystal microbalance with dissipation (QCM-D) experiments was 25 ℃. The flow rate for all solutions was kept at 100 μL/min. The baseline for each measurement was established by flowing deionized water over the quartz crystal for approximately 1 h. Adsorption of LbL film was carried out by alternating the flow of G_n_C_12_ at 5 mg/mL and PSS at 5 mg/mL for 10 min each time. After each adsorption, the system was rinsed for 5 min with deionized water. All films in this study were capped or terminated with G_n_C_12_ and the total number of layer pairs was 5.5. After the final rinse, the films were exposed to either NaCl solution at different concentrations or deionized water at different pH values. Changes in frequency and dissipation from each QCM-D experiment were analyzed using QTools modeling software (Biolin Scientific AB, Gothenburg, Sweden). The surface topography was characterized using a FASTSCAN atomic force microscope (AFM, Bruker Instruments, Madison, WI, USA) in peak-force tapping mode using a silicon cantilever, with a nominal spring constant of 4 N/m and at a scan rate of 1.0 Hz. The cross-section analysis and the roughness (*R*_q_) of adsorbed layers in air were evaluated by the Nanoscope Analysis software (Nanoscope Analysis 2.0, Bruker, Madison, WI, USA). HNTs were dispersed in aqueous solution at a concentration of 0.05 wt.%, which was dropped onto holey carbon film on copper grids, then the excess liquid was removed by filter paper. The morphology of the HNT samples was observed under JEM-1011 transmission electron microscopy (TEM, JEOL, Akishima, Japan) at 200 kV acceleration voltage. The *ζ*-potential values of different LbL systems were measured on a Nano-ZS instrument (Malvern Instruments Ltd., Malvern, UK). The compositions of HNTs samples were determined by a TENSOR-27 Fourier-transform infrared spectroscopy instrument (FTIR, Bruker, Karlsruhe, Germany), with spectra ranging from 4000 to 400 cm^−1^, and the disk shaped samples were obtained by compression molding with KBr. The weight change of HNTs was examined by TG-DTA6300 thermogravimetric analysis (TGA, NSK, Tokyo, Japan) from 30 to 700 °C at a heating rate of 10 °C/min under a N_2_ atmosphere. The percentage of modification of HNTs was calculated using the following equation: modification ratio (%) = (*m*_1_/*m*_2_) × 100%, where *m*_1_ (g) is the weight of organics modified onto HNTs and *m*_2_ (g) is the weight of HNTs. The colloidal viscosity of the PAM gel system was determined using a Bruker Rotary Viscometer (Bruker, Madison, WI, USA).

### 2.3. Fabrication of Methenamine-Loaded HNTs

To entrap methenamine, purified HNTs were mixed as dry powders with a saturated solution of methenamine in ethanol at 1.2 g/mL. A beaker containing methenamine and the HNT suspension was transferred to a vacuum jar, which was then evacuated using a vacuum pump. The methenamine solution was drawn into the internal cavity of HNTs in the presence of negative pressure for 1–2 h [24]. The whole process was repeated 3 times in order to increase the loading efficiency. The nanotubes were washed with distilled water separated by centrifugation. It was reasonable that the methenamine molecules were loaded into the interior cavity of HNTs through the hydrogen bond interactions between their tertiary amine groups and hydroxyl of HNTs.

### 2.4. LbL Assembly of G_n_C_12_/PSS Multilayers, Modifying the Methenamine-Loaded HNTs

LbL assemblies on HNTs were achieved via following the procedures: 0.1 g HNTs loaded with methenamine were first added into 20 mL G_n_C_12_ solution at 10 mg/mL and shaken at 200 rpm at room temperature for 10 min, followed by rinsing with deionized water and centrifuging 3 times. Then, the modified HNTs were dispersed into 20 mL PSS solution (10 mg/mL) for 10 min, followed by washing with deionized water and centrifuging 3 times. The same procedure was repeated 5.5 times to obtain the multilayer of HNT-(G_n_C_12_/PSS)_5.5_.

### 2.5. Controllable Release of Methenamine

The release behavior of methenamine from the HNTs-(G_n_C_12_/PSS)_5.5_ systems was examined by measuring the amount of methenamine released into the bulk solution. Here, 50 mg methenamine-loaded HNT nanocapsules was put into a dialysis tube (MWCO = 2500 Da) and dialyzed against 100 mL distilled water at room temperature. Then, 2 mL solution was taken out from the dialysis solution at set intervals to measure the concentration of the released methenamine from the UV adsorption band at 215.5 nm according to the standard methenamine curve. At the same time, 2 mL distilled water was added to the mixed solution to keep the volume constant. The percentage of the total methenamine released from the nanocapsules was obtained and plotted as a function of time. The cumulative methenamine released was calculated according to Equation (1), in which *M_t_* represents the methenamine amount released in time and *M_∞_* represents the total methenamine amount released.

(1) $$\mathrm{Cumulative}\;\mathrm{methenamine}\;\mathrm{release}\;(\%)=M_{t}/M_{\infty }\times 100\%$$

### 2.6. Crosslinking Evaluation

The crosslinking efficiency of HNTs-(G_n_C_12_/PSS)_5.5_ for the dilute PAM solution was performed by measuring the colloidal viscosity after adding HNTs-(G_n_C_12_/PSS)_5.5_ loaded with the crosslinking agent methenamine at different NaCl concentrations and pH values.

## 3. Results and Discussion

### 3.1. LbL Assembly and Responsiveness to Salt and pH

**Figure 1:**
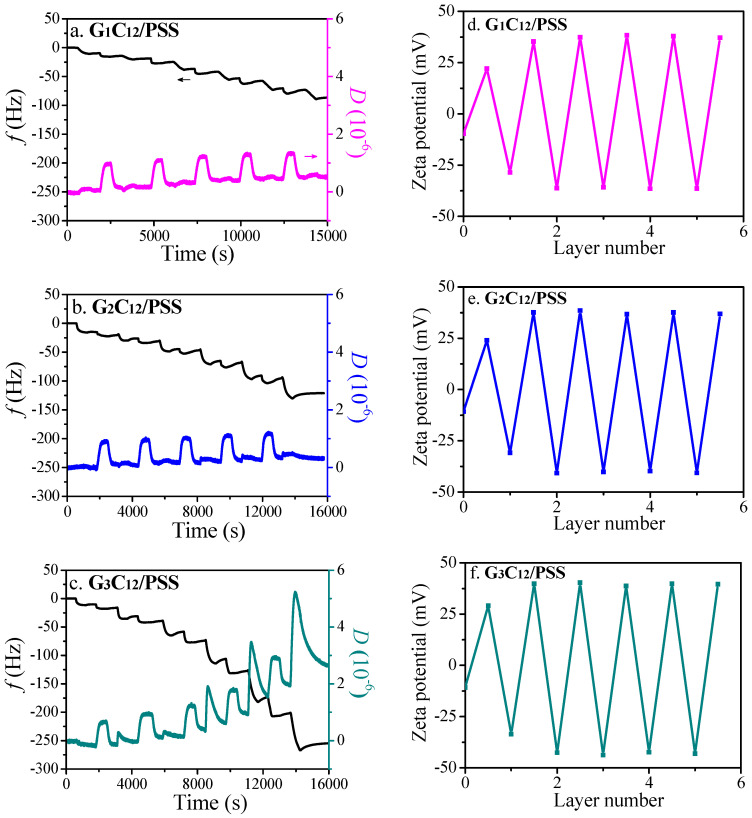
Shifts of frequency and dissipation as a function of time for multilayer assemblies of (**a**) G_1_C_12_/PSS, (**b**) G_2_C_12_/PSS, and (**c**) G_3_C_12_/PSS. Zeta-potentials as functions of the coating number on HNTs modified by (**d**) G_1_C_12_/PSS, (**e**) G_2_C_12_/PSS, and (**f**) G_3_C_12_/PSS multilayers.

The constructing process of G_n_C_12_/PSS LbL thin films and the responsive performances in the external environment were investigated with the QCM-D method in situ. The (G_n_C_12_/PSS)_5.5_ films were assembled by alternately loading G_n_C_12_ and PSS solutions onto SiO_2_-coated quartz crystals, as shown in Figure 1a–c. The final Δ*f* values of the LbL films for G_1_C_12_, G_2_C_12_, and G_3_C_12_ were around −90 Hz, −120 Hz, and −260 Hz, respectively, indicating a dependence on dendrimer generation, which is mainly caused by the increased molecular weight and more adsorbed dendrimer molecules due to the stronger hydrophobic interaction at higher generation [25]. The increase in Δ*D* with the higher generation indicates a softer film with more hydrophobic microdomains in the LbL films. In the same way, LbL nanoshells are fabricated by assembling alternating layers of G_n_C_12_ and PSS onto HNTs, which is demonstrated by zeta potential measurements (Figure 1d–f). A reversal in charge after each adsorbed layer suggests that the corresponding components are sequentially deposited. Accordingly, the zeta potentials of the G_n_C_12_-adsorbed layer reach 25–40 mV due to its positive nature, but turn negative in the case of the PSS adsorbed layer.

**Figure 2:**
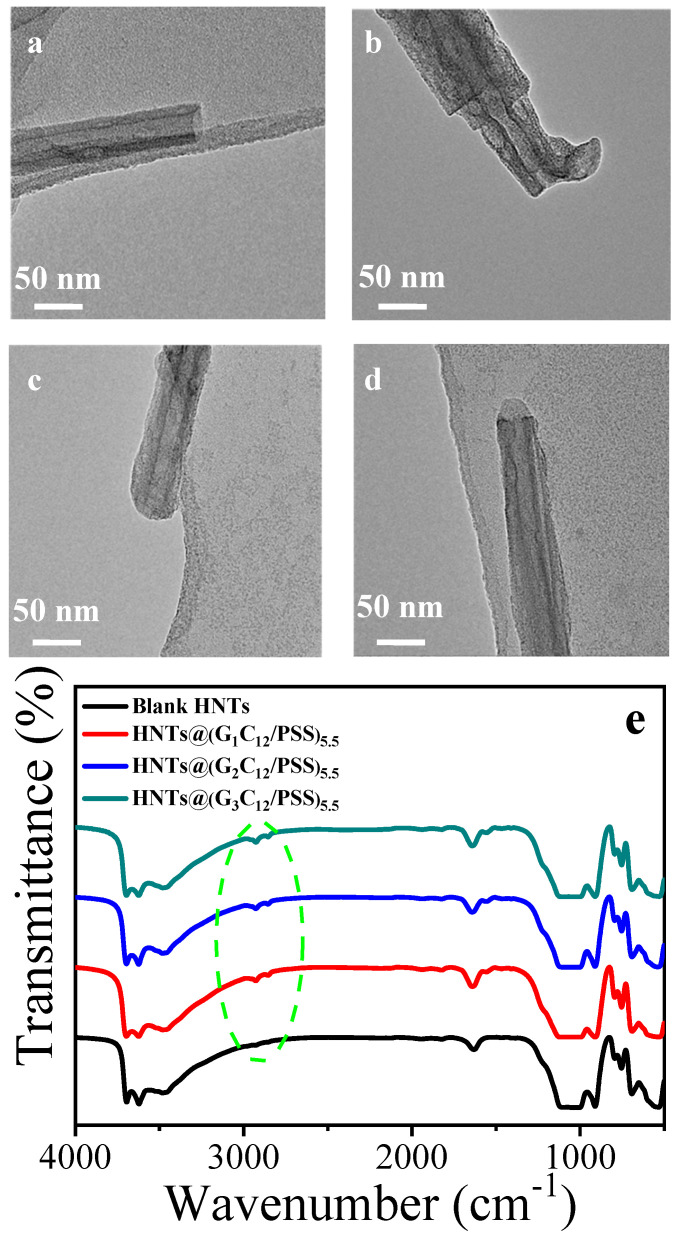
TEM images of (**a**) bare HNTs, (**b**) HNTs@(G_1_C_12_/PSS)_5.5_, (**c**) HNTs@(G_2_C_12_/PSS)_5.5_, (**d**) HNTs@(G3C12/PSS)5.5. (**e**) FTIR spectra of bare HNTs, HNTs@(G_1_C_12_/PSS)_5.5_, HNTs@(G_2_C_12_/PSS)_5.5_, and HNTs@(G_3_C_12_/PSS)_5.5_.

The bare HNT sample had a cylindrical-shaped tubular morphology and open-ended lumen, with an average a diameter of 10–20 nm, as determined by TEM (Figure 2a). In comparison, the rough surface and capped tube ends demonstrated the existence of the LbL nanoshells on HNTs, as shown in Figure 2b–d. The LbL self-assembly of HNTs was also confirmed by FTIR analysis, in which the spectra of HNTs-(G_n_C_12_/PSS)_5.5_ displayed alkyl chain vibration band at around 2980 cm^−1^ (Figure 2e), which was not distinct because of the low ratio of dendrimers in the nanocomposites. Additionally, the peak areas at 3400–3700 cm^−1^ belong to O–H stretching vibration absorption of HNTs; the peak areas of 1000–1100 cm^−1^ belong to Si–O stretching vibration absorption of HNTs. TGA curves (Figure S4) reveal that the residual mass fractions of HNTs with LbL multilayers are lower than pristine HNTs and decrease with increasing dendrimer generations. The above results demonstrate the successful assembly of LbL multilayers on HNTs.

**Figure 3:**
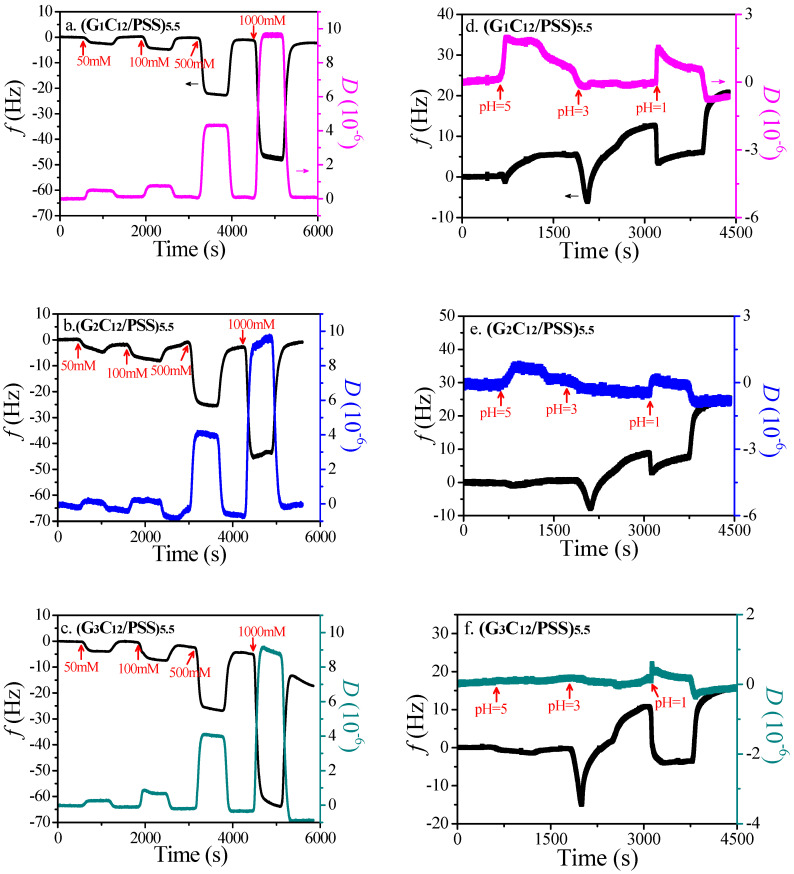
Shifts of frequency and dissipation in response to salt for multilayer assemblies of (**a**) (G_1_C_12_/PSS)_5.5_, (**b**) (G_2_C_12_/PSS)_5.5_, and (**c**) (G_3_C_12_/PSS)_5.5_; and in response to pH for multilayer assemblies of (**d**) (G_1_C_12_/PSS)_5.5_, (**e**) (G_2_C_12_/PSS)_5.5_, and (**f**) (G_3_C_12_/PSS)_5.5_.

After the assembly, the thin films with a roughness of ≈3 nm (Figure S5) were exposed to the NaCl solution at a given concentration under dynamic flow, then after rinsing the films were incubated in another brine at a higher ionic strength as a subsequent rinsing step. Figure 3a–c shows the decreasing Δ*f* values and increasing Δ*D* values when the salt concentration increases from 50 mM to 1000 mM. Compared with the salt effect on the bare SiO_2_ substrate (Figure S6), this indicates that the modified films become more viscoelastic and swell during the salt flushing process. This can be attributed to the adsorption of counterions into the interior of the LbL film, resulting in electrostatic screening on the ion pairs of G_n_C_12_ and PSS and concomitant film swelling [26,27]. A similar trend can be observed with higher dendrimer generation and with a higher amount of positively charged amino groups. Therefore, with either higher salt concentration or higher dendrimer generation, LbL films tend to swell more, which may result in a slower release of guest molecules.

Interestingly, when lower pH values are introduced to the LbL films (Figure 3d–f), there is an increase in frequency and a decrease in dissipation, indicating a desorption of films, which agrees with the decreasing surface roughness at lower pH values (Figure S7) compared with those in a neutral environment (Figure S5). This can be attributed to the higher degree of the protonation of PSS [27,28] and the weaker electrostatic interactions of the PSS-dendrimer at lower pH values. With higher dendrimer generation, there is also a large reduction of electrostatic interactions between the PSS and dendrimer, resulting in higher desorption of films. Therefore, with either a higher acidic environment or higher dendrimer generation, LbL films tend to gradually detach from the substrate, which may result in a quicker release of guest molecules.

### 3.2. Release Performance of the Loaded Methenamine

**Figure 4:**
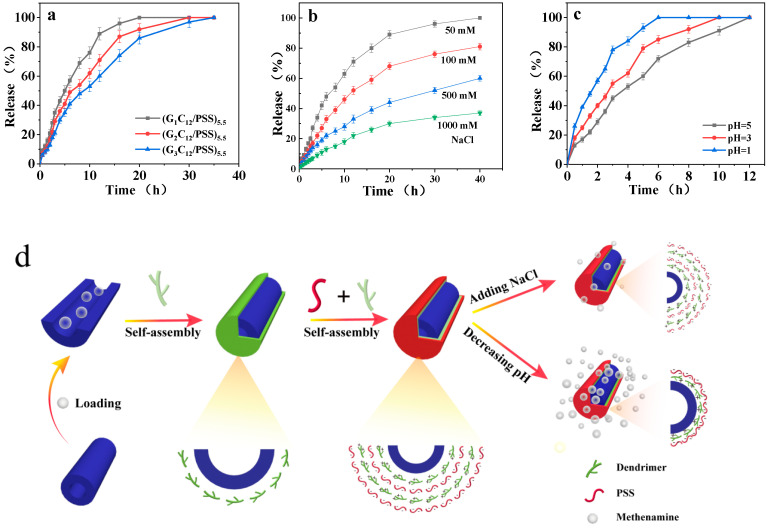
Release profiles of methenamine from different systems: (**a**) LbL assemblies in the presence of different dendrimer generations, (**b**) in the presence of NaCl at different concentrations in HNTs@(G_3_C_12_/PSS)_5.5_ systems, (**c**) under different pH values, and in the presence of HNTs@(G_1_C_12_/PSS)_5.5_. (**d**) Schematic illustration of HNTs@(G_n_C_12_/PSS)_5.5_ with salinity–pH dual-responsive release performances.

The dependence of methenamine release on the dendrimer generation, salinity, and pH condition was investigated to evaluate the performance of the salt and pH dual-responsive HNTs-(G_n_C_12_/PSS)_5.5_ nanocomposites. Figure 4a displays the cumulative methenamine release from HNTs-(G_n_C_12_/PSS)_5.5_ with different dendrimer generations in deionized water. The bare HNTs exhibit a rapid release of 80% in 2 h, whereas the encapsulation via LbL assemblies eliminates the burst release to 30–40 h. The guest molecules are released slowly with the higher dendrimer generation due to the formation of thicker nanoshells in such cases. As the salt concentration arises, the release rate is lower in the presence of HNTs-(G_3_C_12_/PSS)_5.5_, as shown in Figure 4b. This can be explained by the fact that the influx of salt ions into the shell can shield the dendrimer–PSS ion pairs, inducing the film swelling and inhibiting the release of methenamine to some extent. At lower pH values, the release rate is higher, taking HNTs-(G_1_C_12_/PSS)_5.5_ as an example (Figure 4c). This shows the pH-sensitive nature of LbL nanoshells, which cause the protonation of PSS and weaken the electrostatic attraction between the dendrimer and PSS, resulting in the gradual desorption of films and the quicker release of methnamine. The results demonstrate that the series of HNTs-(G_n_C_12_/PSS)_5.5_ nanocomposites can release cargoes in several hours and up to several days in response to salinity and pH stimuli, as illustrated in Figure 4d.

### 3.3. Crosslinking Efficiency in PAM System

**Figure 5:**
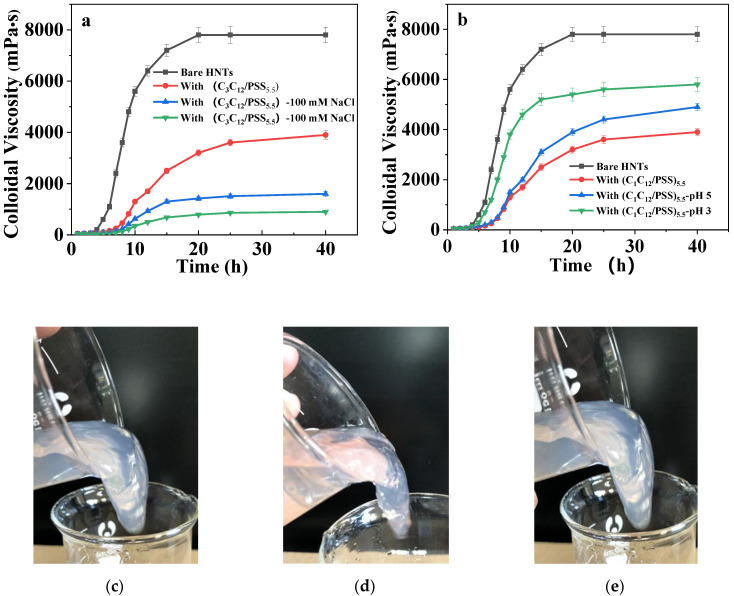
The colloidal viscosity–time profiles of polyacryamide solutions under different conditions: (**a**) in the presence of NaCl at different concentrations and HNTs@(G_3_C_12_/PSS)_5.5_; (**b**) under different pH values and in the presence of HNTs@(G_1_C_12_/PSS)_5.5_; (**c**) PAM gel without HNTs; (**d**) PAM gel in the presence of NaCl at different concentrations in HNTs@(G_3_C_12_/PSS)_5.5_ systems; (**e**) PAM gel at pH = 5 in the presence of HNTs@(G_1_C_12_/PSS)_5.5_.

The practical crosslinking experiment was performed to evaluate the crosslinking efficiency of the hybrid controllable system loaded with methenamine into dilute PAM solutions. The colloidal viscosity of the PAM solution increases to a maximum value of 8000 mPa·s at 20 h in the presence of bare HNTs (Figure 5a), showing a gel state and a typical “tongue thrust” phenomenon (Figure 5c and Movie S1). For the methenamine loaded with the modified HNTs, the colloidal viscosity decreases notably compared with the bare HNTs, the maximum viscosity value of 4000 mPa·S reaches 40 h, and the system is not accompanied with the gel formation. The introduction of NaCl to the system dramatically decreases the crosslinking rate of the PAM solution, in which the colloidal viscosity is merely 1400 mPa·S and 800 mPa·S at salinity values of 100 mmol/L and 500 mmol/L after 60 h, respectively. Furthermore, the gel formation under 500 mmol/L NaCl occurs after about 15 days (Figure 5d and Movie S2) due to the decreasing release rate of methenamine triggered by salt. The lower pH in systems can speed up the gel formation (Figure 5b) compared with the pure HNTs-(G_1_C_12_/PSS)_5.5_ system, as indicated by the increased colloidal viscosity. The viscosity reaches 5500 mPa·S and 4000 mPa·S at pH = 5 and pH = 3, respectively, while the PAM gel forms at ≈3 days for pH = 5, as shown in Figure 5e and Movie S3. The release rate of methenamine is accelerated in lower pH conditions due to the desorption of films. From all the results above, a series of dual-responsive nanocapsules based on layer-by-layer assemblies are fabricated, which is predicted to precisely control the release of crosslinking agents and the formation of gelation from a few hours to a dozen days.

## 4. Conclusions

A series of salt- and pH-responsive controlled release systems, in which amphiphilic dendrimers and poly(styrene sulfonic acid) sodium polymers self-assemble on halloysite nanotubes via the LbL technique, which can control the release rate of methenamine and the crosslinking rate of PAM. Methenamine is released slowly at higher NaCl concentrations due to the swelling of dendrimer–PSS films because of the electrostatic shielding effect, and rapidly at lower pH values because of the desorption of the films, which is caused by the weakened interlayer interaction. Accordingly, the formation time of the PAM gel can be perfectly controlled from a few hours to a dozen days, providing a potential way to achieve “precise” profile control. In summary, a new type of dual-responsive nanocapsule has been developed to encapsulate guest molecules and control the release on demand, which is a candidate for diverse applications in environmentally dependent nanostructures.

## Supplementary Materials

The following are available online at https://www.mdpi.com/1996-1944/13/16/3479/s1: Scheme S1: The synthesis pathway of amphiphilic dendrimer G_1_C_12_, Figure S1: Characterization data of G_1_C_12_ by ^1^H NMR, Figure S2: Characterization data of G_2_C_12_ by ^1^H NMR, Figure S3: Characterization data of G_3_C_12_ by ^1^H NMR, Figure S4: TGA curves of pristine HNTs, HNTs@(G_1_C_12_/PSS)_5.5_, HNTs@(G_2_C_12_/PSS)_5.5_, and HNTs@(G_3_C_12_/PSS)_5.5_ under N_2_, Figure S5: The 5 µm × 5 µm AFM images: (a) (G_1_C_12_/PSS)_5.5_; (b) (G_2_C_12_/PSS)_5.5_; (c) (G_3_C_12_/PSS)_5_._5_, Figure S6: Shifts of frequency and dissipation for multilayers in response to different salt concentration as a function of time for bare silica substrate, Figure S7: The 5 µm × 5 µm AFM images of adsorbed (G_1_C_12_/PSS)_5.5_ films in response to different pH buffer solutions: (a) pH = 5; (b) pH = 3; (c) pH = 1, Movie S1: PAM gel at pH = 5 in the presence of HNTs@ (G_2_C_12_/PSS)_5.5_, Movie S2: PAM gel in the presence of NaCl. Movie S3: PAM gel without HNTs.
