# Supplementary material for: Dual-Responsive Nanotubes Assembled by Amphiphilic Dendrimers: Controlled Release and Crosslinking
Source: Materials (Basel). 2020 Aug 7;13(16):3479. doi: 10.3390/ma13163479 (PMC7475864; doi:10.3390/ma13163479)
Supplement: Supplementary file 1 [file materials-13-03479-s001.zip › materials-860507-supplementary.docx]

Supplementary Materials: Dual-Responsive Nanotubes Assembled by Amphiphilic Dendrimers: Controlled Release and Crosslinking

Minghui Zhang ^1,2^, Hui Yang ^1,^*, Jiazhong Wu ^3^, Siyu Yang ^3^, Danfeng Yu ^4^, Xu Wu ^4^, Aiqing Ma ^5^, Keji Sun ^5^ and Jinben Wang ^1^

Minghui Zhang ^1,2^, Hui Yang ^1,^*, Jiazhong Wu ^3^, Siyu Yang ^3^, Danfeng Yu ^4^, Xu Wu ^4^, Aiqing Ma ^5^, Keji Sun ^5^ and Jinben Wang ^1^

^1^ CAS Key Lab of Colloid, Interface and Chemical Thermodynamics, Institute of Chemistry, Chinese Academy of Sciences, Beijing 100190, China; [zhangminghui@iccas.ac.cn](javascript:void(0);) (M.Z.); jbwang@iccas.ac.cn (J.W.)

^2^ University of Chinese Academy of Sciences, Beijing 100049, China

^3^ State Key Laboratory of Enhanced Oil Recovery, Research Institute of Petroleum Exploration and Development of PetroChina, Beijing 100083, China; [wujiazhong@petrochina.com.cn](javascript:void(0);) (J.W.); [yangsiy@petrochina.com.cn](javascript:void(0);) (S.Y.)

^4^ Department of Chemistry and Chemical Engineering, Guangzhou University, Guangzhou 510006, China; [ccyudanfeng@gzhu.edu.cn](javascript:void(0);) (D.Y.); [xuwu@gzhu.edu.cn](javascript:void(0);) (X.W.)

^5^ Petroleum Engineering Technology Research Institute, Shengli Oilfield Branch, China Petrochemical Corporation LTD, Dongying 257000, China; [maq7979@163.com](javascript:void(0);) (A.M.); [skj502@sina.com](javascript:void(0);) (K.S.)

* Correspondence: [yanghui@iccas.ac.cn](javascript:void(0);); Tel: +8610-62523395; Fax: +8610-62523395

G_n_C_12_ was synthesized using 0.5 G, 1.5 G, and 2.5 G Polyamindoamine (PAMAM) as the core material. The tertiary amine was introduced by reaction with N,N-Dimethyl-1,2-ethanediamine. Then, the tertiary amine groups reacted with 1-bromododecane to form the quaternary ammonium compound. All the products were recrystallized by acetone or ethanol repeatedly and characterized by ^1^H NMR. Taking G_1_C_12_ for example, a scheme of the reaction is shown in Scheme S1.


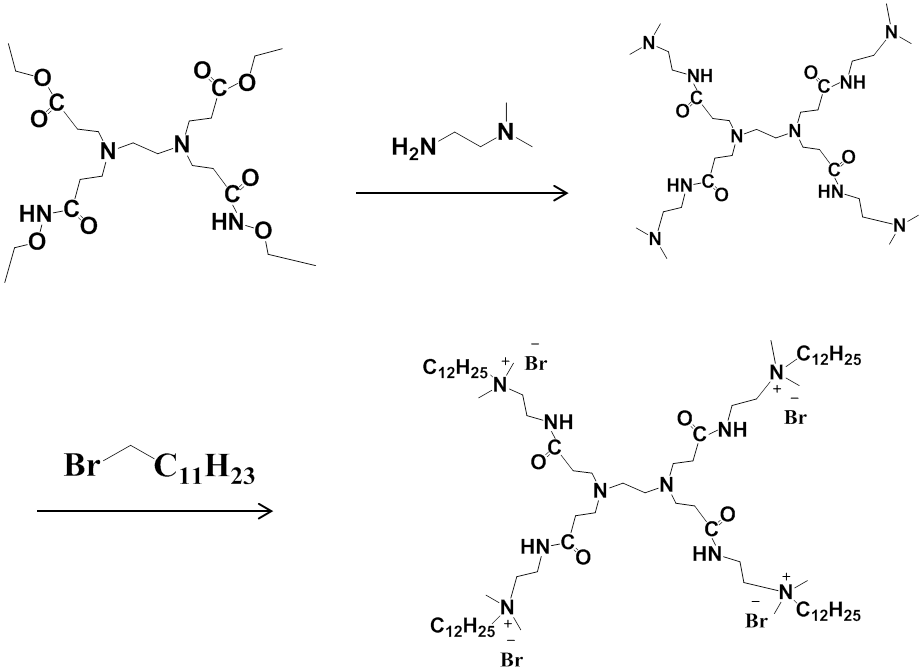


**Scheme S1.** The synthesis pathway of the amphiphilic dendrimer G_1_C_12_.

**G_1_C_12_**: ^1^H NMR (400 MHz, CDCl_3_) δ 9.63–8.10 (m, 4H), 3.90 (t, *J* = 84.9 Hz, 16H), 3.47 (d, *J* = 46.1 Hz, 32H), 3.19 (s, 4H), 3.09–2.79 (m, 8H), 2.78–2.32 (m, 8H), 1.75 (s, 8H), 1.25 (s, 72H), 0.88 (t, *J* = 6.7 Hz, 12H).

**G_2_C_12_**: ^1^H NMR (400 MHz, CDCl_3_) δ 9.45–8.39 (m, 8H), 8.05 (s, 4H), 3.74 (s, 32H), 3.51 (s, 24H), 3.32 (d, *J* = 16.6 Hz, 48H), 2.46–2.75 (t, 40H), 1.74–2.01 (s, 36H), 1.25–1.35 (m, 144H), 0.88 (t, *J* = 6.9 Hz, 24H).

**G_3_C_12_**: ^1^H NMR (400 MHz, CDCl_3_) δ 8.72 (d, *J* = 60.5 Hz, 16H), 8.09 (s, 8H), 7.93 (s, 4H), 3.59 (d, *J* = 111.3 Hz, 120H), 3.31 (d, *J* = 38.3 Hz, 96H), 2.75 (s, 56H), 2.62–2.21 (m, 80H), 1.74 (s, 36H), 1.28 (d, *J* = 41.4 Hz, 288H), 0.86 (t, *J* = 6.6 Hz, 48H).


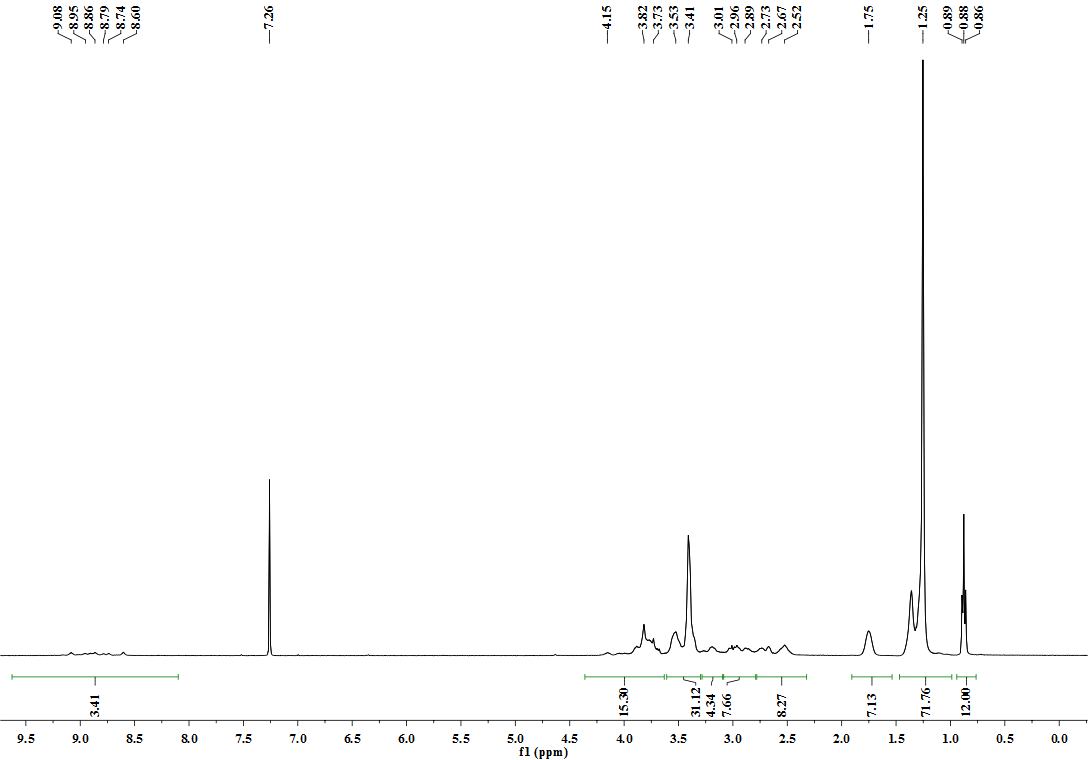


**Figure S1.** Characterization data of G_1_C_12_ using ^1^H NMR.

**
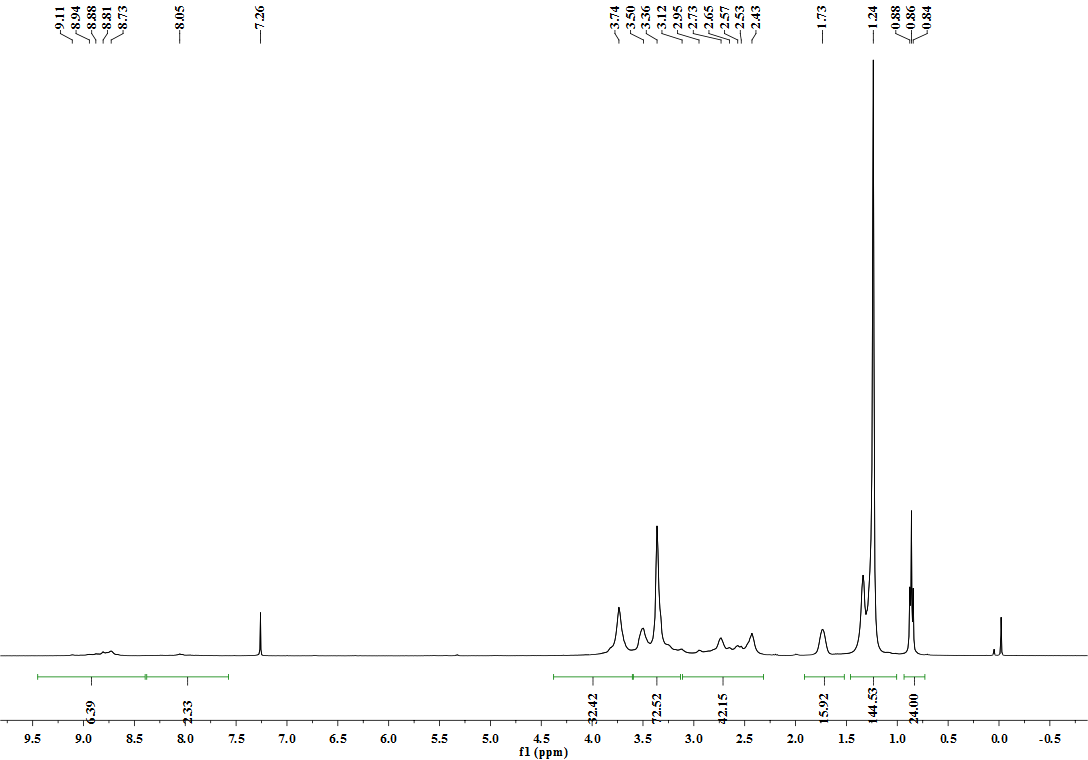
Figure S2.** Characterization data of G_2_C_12_ using ^1^H NMR.


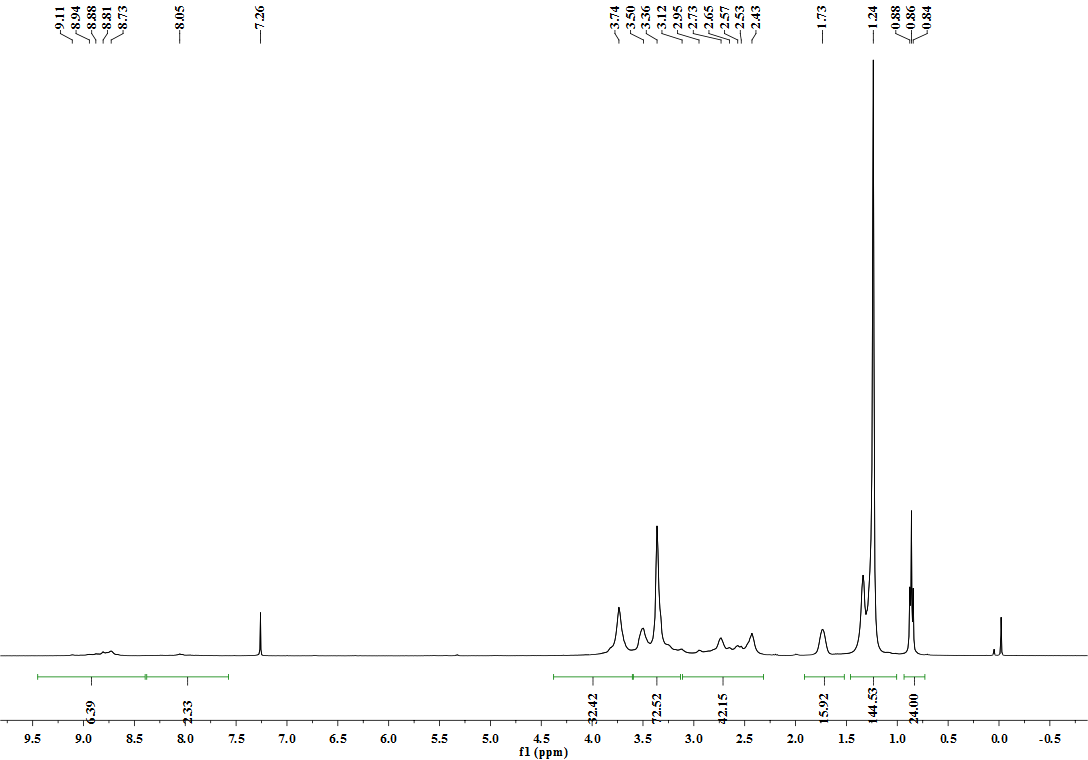


**Figure S3.** Characterization data of G_3_C_12_ using ^1^H NMR.

**Figure S4.** TGA curves of pristine HNTs, [HNTs@(G_1_C_12_/PSS)_5.5_](mailto:HNTs@(G1C12/PSS)5.5), HNTs@(G_2_C_12_/PSS)_5.5_, and HNTs@(G_3_C_12_/PSS)_5.5_ under N_2_.


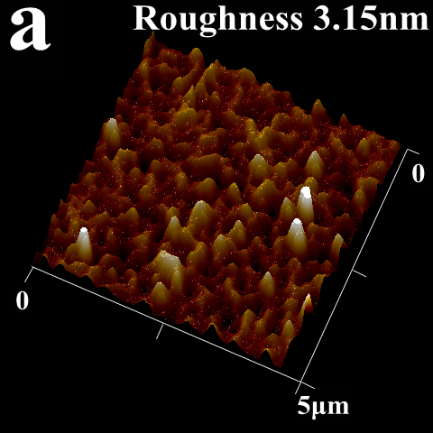

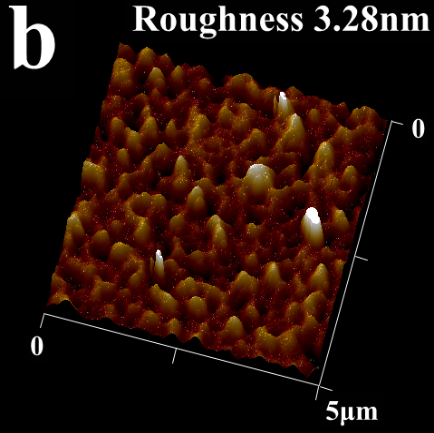


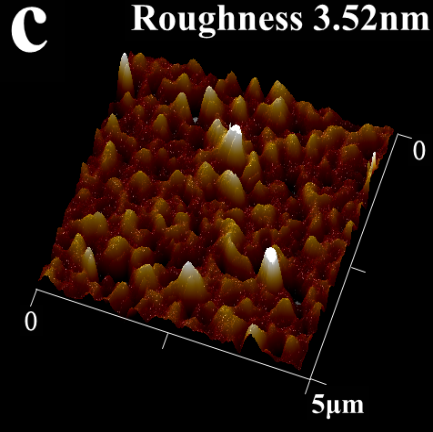


**Figure S5.** The 5 µm × 5 µm AFM images: (a) (G_1_C_12_/PSS)_5.5_; (b) (G_2_C_12_/PSS)_5.5_; (c) (G_3_C_12_/PSS)_5_._5_.

**Figure S6.** Shifts of frequency and dissipation for multilayers in response to different salt concentrations as a function of time for bare silica substrate.


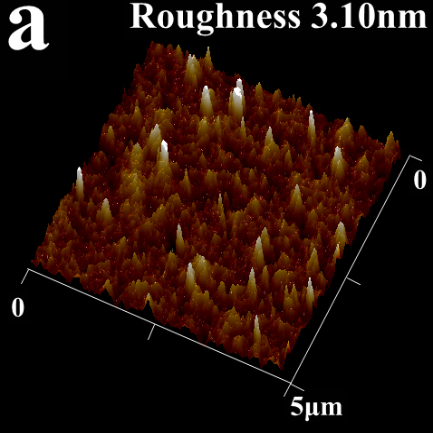

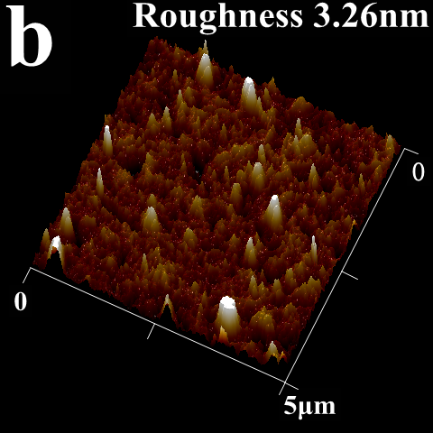


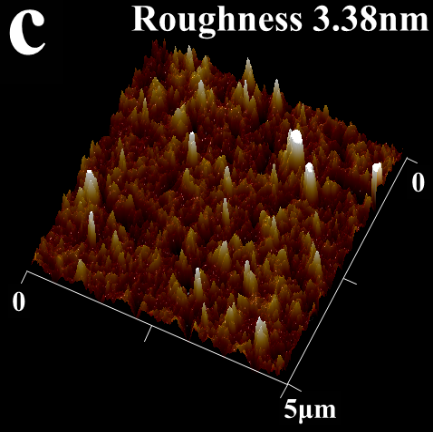


**Figure S7.** The 5 µm × 5 µm AFM images of adosorbed (G_1_C_12_/PSS)_5.5_ films in response to different pH buffer solutions: (a) pH = 5; (b) pH = 3; (c) pH = 1.
